# Acupuncture and related techniques for restless legs syndrome: A protocol for systematic review and meta-analysis

**DOI:** 10.1097/MD.0000000000022205

**Published:** 2020-09-25

**Authors:** Jie Xiang, Honglian Li, Jun Xiong, Fanghui Hua, Shouqiang Huang, Yunfeng Jiang, Hailiang Qiang, Fan Xie, Min Wang

**Affiliations:** a Jiangxi University of Traditional Chinese Medicine; b Haiyang People's Hospital of Shandong Province, Haiyang; c Affiliated Hospital of Jiangxi University of Traditional Chinese Medicine, Nanchang, PR China.

**Keywords:** acupuncture, protocol, restless legs syndrome, systematic review

## Abstract

**Background:**

Restless legs syndrome (RLS) is a common sensory disorder of the nervous system, which often affects the sleep quality of patients. Acupuncture and related techniques are increasingly used to treat neurological diseases, but their efficacy and safety for RLS are yet to be established. The purpose of this study is to summarize the effectiveness and safety of acupuncture and related techniques for RLS.

**Methods:**

We will conduct a comprehensive data retrieval, and the electronic databases will include PubMed, Embase, Cochrane Library, WangFang Database, China National Knowledge Infrastructure, Chinese Scientific Journal Database, Chinese Biomedical Literature Database, from establishment to October 2020. We will also manually search unpublished studies and references, and contact lead authors. Randomized clinical trials (RCTs) of acupuncture and related techniques for RLS will be included. The outcomes of interest include: The total effective rate and International Restless Leg Syndrome rating scale (IRLS), Pittsburgh Sleep Quality Index (PSQI), Hamilton Anxiety Scale (HAMA), Hamilton Depression Scale (HAMD), adverse events, quality of life. To assess the methodological quality, we will use the Cochrane risk assessment tool. RevMan 5.3.5 software will be used to conduct data synthesis. The evidence quality of each outcome will be appraised according to Grades of Recommendation, Assessment, Development, and Evaluation (GRADE).

**Results:**

The results will be published in a peer-reviewed journal.

**Conclusion:**

This study will provide a high-quality evidence to evaluate the efficacy and adverse reactions of acupuncture and related techniques for RLS.

**PROSPERO registration number:**

CRD42020157957.

## 1 Introduction

Restless legs syndrome (RLS, also called Willis-Ekbom disease), is a common sensory disorder of the nervous system, which is mainly manifested by patients desire for legs activity at night or at rest, accompanied by discomfort, and the symptoms are relieved after the activity. Diagnosis should be distinguished from certain diseases (such as muscle pain, varicose veins, arthritis, lower extremity edema).^[1]^ There is currently no objectively available tests to diagnose RLS, mainly based on subjective symptom descriptions.^[2]^

In terms of prevalence, North America and Europe are the highest, estimated at between 5.5% and 11.6%, and relatively lower in Asia, estimated at between 1.0% and 7.5%.^[3]^ Women over 35 years old are twice as likely to suffer from this disease as men, and the prevalence of children is 2%.^[4,5]^ The prevalence of RLS is mainly related to the following factors: genetics, iron deficiency, kidney disease, pregnancy, Parkinson, certain drugs (such as antidepressants, antipsychotics, histamine receptor blockers), smoking, drinking, caffeine, etc.^[6,7]^ Previous studies have shown that family history of more than 60% of the patients with RLS.^[6]^ Other studies have shown that the risk of RLS in patients with iron deficiency anemia is 5 to 6 times that of the general population, and the prevalence of patients with end-stage renal disease is 6.6% to 68%.^[8,9]^

The pathogenesis of RLS is still unclear. Existing studies indicate that it may be related to dopamine transport disorders, certain genes, neurotransmitter and neural pathway abnormalities, and neuroanatomical abnormalities.^[10–13]^ A large number of studies have shown that the quality of life of RLS patients is affected, and 75% of patients have sleep disturbances, which can affect work in serious cases.^[14–17]^ Long-term RLS may affect cardiovascular disease, diabetes, and cause autonomic disorders, which may be related to sympathetic nerve excitement.^[18,19]^ Psychological distress is also more common in patients with RLS.^[20]^ Studies on male patients with RLS suggest that RLS is associated with sexual dysfunction.^[21]^

At present, α2δ ligands and dopamine agonists are recommended as the first-line drugs for the treatment of RLS. Second-line drugs are opioids, benzodiazepines and iron.^[22]^ These drugs are selected according to the individuation of patients, which has been proved to be effective, but followed by some adverse consequences. According to the survey,^[23]^ 76% of patients using dopamine agonists developed augmentation (delayed exacerbation of symptoms) over time. Some drugs are dependent, causing dizziness, drowsiness, nausea, and so on.^[24]^ Because the drugs are not applicable in some cases and the side effects can not be ignored, some non-pharmacologic treatments with less side effects may be the methods for the treatment of RLS.

Acupuncture is a kind of external treatment, is a traditional Chinese treatment.^[25]^ It has been proven to be an effective and well-tolerated therapy for the treatment of neurological diseases.^[26,27]^ Studies have shown that its side effects are far less than dopamine agonists.^[28,29]^ Therefore, it is possible that acupuncture of RLS is effective and safe. Acupuncture and related techniques for the treatment of RLS include acupuncture, warming acupuncture, electroacupuncture, moxibustion and so on.

At present, there are 3 published systematic reviews of acupuncture of RLS.^[30–32]^ One of them included only 2 studies before 2007, and the conclusion was insufficient. The other 2 systematic reviews were published in Chinese. They did not conduct a comprehensive literature search, and the outcomes were few. The conclusions could not truly reflect the effectiveness and safety of acupuncture treatment for RLS. Therefore, we consider that it is necessary to re-conduct a systematic review. We will strictly abide by the method of systematic review in order to provide more reliable evidence for doctors and researchers, as well as more reasonable treatment for RLS patients.

## 2 Methods

### 2.1 Study registration

Our protocol has been registered in PROSPERO (CRD42020157957). This report will be conducted according to the Preferred Reporting Items for Systematic reviews and Meta-Analysis Protocols (PRISMA-P).^[33]^ The changes will be described in our full review.

### 2.2 Inclusion criteria

#### 2.2.1 Type of studies

This study will include all relevant randomized clinical trials (RCTs) without language restrictions.

#### 2.2.2 Type of participants

RLS patients who meet the diagnostic criteria will be included, regardless of age, gender, race.

#### 2.2.3 Type of interventions

Acupuncture and related techniques will choose warming acupuncture, electroacupuncture, moxibustion, manual acupuncture, ear acupuncture, acupressure, etc. Combination with other conventional therapies (e.g., medication/drugs) will also be allowed.

#### 2.2.4 Type of comparators

Conventional treatments, placebo, no treatment or sham acupuncture will be as comparators. However, the comparisons between acupuncture and related techniques will be excluded.

#### 2.2.5 Types of outcome measures

##### 2.2.5.1 Primary outcomes

The total effective rate and international restless leg syndrome rating scale (IRLS).

##### 2.2.5.2 Secondary outcomes

1. Pittsburgh sleep quality index (PSQI).
2. Hamilton anxiety scale (HAMA).
3. Hamilton Depression Scale (HAMD).
4. Adverse events.
5. Quality of life.

### 2.3 Exclusion criteria

Studies that are repeatedly published and necessary information cannot be obtained in various ways will be excluded.

### 2.4 Search methods for identification of studies

We will conduct a comprehensive data retrieval, and the electronic databases will include PubMed, Embase, Cochrane Library, WangFang Database, China National Knowledge Infrastructure, Chinese Scientific Journal Database, Chinese Biomedical Literature Database, from establishment to October 2020. We will also manually search unpublished studies and references. The specific search strategy of Pubmed is provided in Table 1.

**Table 1:**
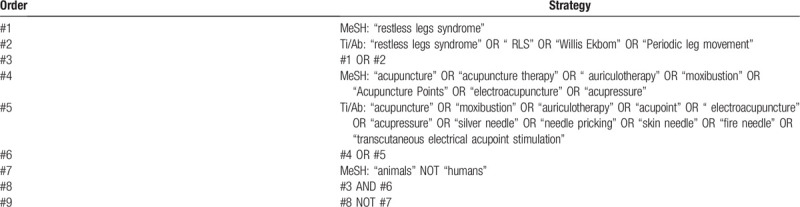
The search strategy for PubMed.

### 2.5 Studies selection

**Figure 1:**
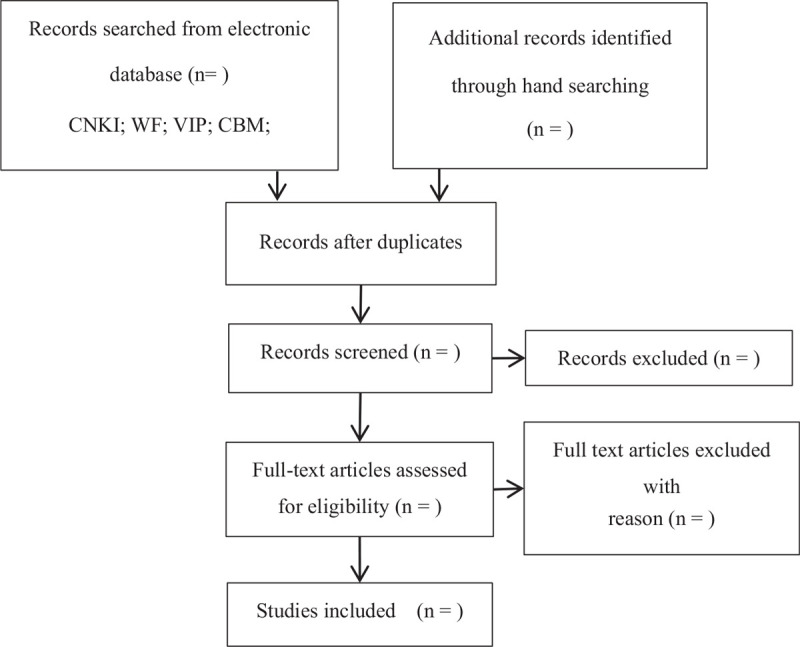
Flowchart of literature selection.

NoteExpress3.2.0 software will eliminate duplicate studies from all the obtained literatures. The unqualified studies in the remaining articles will be eliminated by 2 reviewers by reading the title and abstract. Then, 2 reviewers will read the full text to determine the final included studies. If the significant information of the article is incomplete, we will contact the author. In all the processes, the researchers will operate independently. When 2 reviewers have disagreements, the decision will be made by the third researcher. The above process is presented in the flowchart (Fig. 1).

### 2.6 Data extraction and management

We will establish a data extraction table, which will be used by 2 researchers to extract data from qualified literature. The specific contents will include: author, publication time, participant characteristics, intervention (s), comparison (s), outcome (s), adverse events and some relevant features. If the significant information of the article is incomplete, we will contact the author. In case of disagreements, the third researcher will be consulted.

### 2.7 Assessment of the methodological quality

The Cochrane risk assessment tool will be used by us to evaluate the methodological quality of qualified RCTs.^[34]^ It includes 7 items: random sequence generation, allocation concealment, blinding of participants and caregivers, blinding of outcome assessors, incomplete outcome data, selective outcome reporting, and other bias. The evaluation result of each item will be “high risk”, “low risk”, or “unclear risk”. The assessment will be completed by 2 reviewers, and disagreements will be handed over to the third reviewer for the final decision.

### 2.8 Measures of treatment effect

Mean difference (MD) or standard mean difference (SMD) will be used for continuous outcomes with 95% confidence intervals (CIs). Dichotomous outcomes will be summarized by risk ratio (RR) with 95% CIs.

### 2.9 Dealing with missing data

We will contact the author by phone or email to obtain complete information. If we cannot obtain that missing data, the analysis will be performed according to the available data. Besides, we will consider the potential impact of missing data for our studies. Otherwise, we will rule out the study.

### 2.10 Assessment of heterogeneity

We will use chi-square test and *I*^2^ value to verify heterogeneity. When *P* < .1, *I*^2^ > 50%, there is significant heterogeneity between studies; otherwise, heterogeneity is acceptable.

### 2.11 Data synthesis

Data synthesis will be completed using RevMan5.3.5 software (Copenhagen: The Nordic Cochrane Centre, The Cochrane Collaboration, 2014). When *I*^2^ < 50%, we will choose the fixed effects model; Otherwise, the random effects model will be selected. The forest plots will present the results of the meta-analyses. We will conduct descriptive analysis, when the results are not suitable for consolidation. When more than 10 studies are included, we will use the funnel plot to assess publication bias.

### 2.12 Subgroup analysis

If necessary, subgroup analyses will be performed according to the different types of participant characteristics, treatment methods, treatment frequency, and so on.

### 2.13 Sensitivity analysis

When there is significant heterogeneity, we will conduct a sensitivity analysis. We will determine the robustness of the results by excluding low-quality studies.

### 2.14 Summary of evidence

We will evaluate the evidence quality of each outcome based on Grades of Recommendation, Assessment, Development, and Evaluation (GRADE).^[35]^ Two reviewers will conduct independent evaluation, and if there are disagreements, the third author will give the decision.

### 2.15 Ethics and dissemination

In this study, no individual data from participants will be involved, so ethics approval is not required. This systematic review will be published through peer-reviewed journal.

## 3 Discussion

RLS is a disease that has a great influence on patients quality of life. At present, the internationally recommended treatment is pharmacotherapy, but if patients use drugs for a long time, the side effects cannot be ignored. RCTs have proved that acupuncture is effective in treating RLS with little side effects. Therefore, we hope that this study can provide a high level of evidence-based evidence for the effectiveness and safety of acupuncture and related techniques in the treatment of RLS, and guide clinical decision-making.

## Author contributions

**Conceptualization:** Jie Xiang, Jun Xiong.

**Data curation:** Jie Xiang, Fanghui Hua, Shouqiang Huang.

**Formal analysis:** Yunfeng Jiang, Hailiang Qiang, Min Wang.

**Investigation:** Jie Xiang, Jun Xiong.

**Methodology:** Honglian Li, Jun Xiong, Fanghui Hua, Shouqiang Huang.

**Software:** Yunfeng Jiang, Hailiang Qiang, Min Wang.

**Supervision:** Jun Xiong, Fan Xie.

**Writing – original draft:** Jie Xiang, Jun Xiong, Fanghui Hua, Shouqiang Huang.

**Writing – review & editing:** Honglian Li, Yunfeng Jiang, Fan Xie, Min Wang.
